# Electrochemical Synthesis of Polypyrrole, Reduced Graphene Oxide, and Gold Nanoparticles Composite and Its Application to Hydrogen Peroxide Biosensor

**DOI:** 10.3390/nano6110220

**Published:** 2016-11-21

**Authors:** Baoyan Wu, Na Zhao, Shihua Hou, Cong Zhang

**Affiliations:** 1 MOE Key Laboratory of Laser Life Science & Institute of Laser Life Science, College of Biophotonics, South China Normal University, Guangzhou 510631, China; wubaoyan@scnu.edu.cn (B.W.); zhaona5251@163.com (N.Z.); 2 School of Electronic and Information Engineering, South China University of Technology, Guangzhou 510640, China; 3 The Key Laboratory of Bioactive Materials Ministry of Education, College of Life Sciences, Nankai University, Tianjin 300071, China; zhangcong0672@163.com

**Keywords:** electrodeposition, polypyrrole, reduced graphene oxide, gold nanoparticles, hydrogen peroxide biosensor

## Abstract

Here we report a facile eco-friendly one-step electrochemical approach for the fabrication of a polypyrrole (PPy), reduced graphene oxide (RGO), and gold nanoparticles (nanoAu) biocomposite on a glassy carbon electrode (GCE). The electrochemical behaviors of PPy–RGO–nanoAu and its application to electrochemical detection of hydrogen peroxide were investigated by cyclic voltammetry. Graphene oxide and pyrrole monomer were first mixed and casted on the surface of a cleaned GCE. After an electrochemical processing consisting of the electrooxidation of pyrrole monomer and simultaneous electroreduction of graphene oxide and auric ions (Au^3+^) in aqueous solution, a PPy–RGO–nanoAu biocomposite was synthesized on GCE. Each component of PPy–RGO–nanoAu is electroactive without non-electroactive substance. The obtained PPy–RGO–nanoAu/GCE exhibited high electrocatalytic activity toward hydrogen peroxide, which allows the detection of hydrogen peroxide at a negative potential of about −0.62 V vs. SCE. The amperometric responses of the biosensor displayed a sensitivity of 40 µA/mM, a linear range of 32 µM–2 mM, and a detection limit of 2.7 µM (signal-to-noise ratio = 3) with good stability and acceptable reproducibility and selectivity. The results clearly demonstrate the potential of the as-prepared PPy–RGO–nanoAu biocomposite for use as a highly electroactive matrix for an amperometric biosensor.

## 1. Introduction

Electrochemical sensors can be considered as one of the most popular applications of nanoparticles due to their high catalytic activities and electron transfer, and easy modification by a wide range of biomolecules and chemical ligands [1]. Electrodes modified with such nanoparticles can reduce overpotential and exhibit good electrocatalysis [2]. Among these nanomaterials, gold nanoparticles (nanoAu) was one of the most widely used metal nanoparticles, which can act as tiny conduction centers and can facilitate the transfer of electrons [3]. Reduced graphene oxide (RGO) is another material that has been widely used in electrochemical analyses, due to its high electrical conductivity and high effective surface area [4,5]. Meanwhile, polypyrrole (PPy)—a type of conducting organic polymer—has also attracted enormous research interest for its widespread applications in electrochemical biosensors thanks to its interesting redox behavior and good environmental stability [6,7].

Most notably, nanocomposites combining two or several different components are expected to further improve the deficient characteristics of each component, a concept which has led to the development of various electrochemical sensors [8,9]. In this context, to date, several studies have been carried out in the area of PPy, RGO and nanoAu composites. For example, Song et al. reported a nanocomposite comprised of nanoAu on the surface of RGO via facile wet-chemical routes by using PPy as a linker, and the composite exhibited excellent electrocatalytic property toward O_2_ reduction [10]. Zhang et al. reported an electrochemical sensor for levofloxacin based on a molecularly imprinted PPy–graphene–nanoAu modified electrode, reducing auric ions (Au^3+^) by sodium borohydrate at 80 °C [11]. Li et al. reported a nanocomposite of nanoAu, PPy and RGO sheets based on electrochemical deposition of RGO with PPy and the introduction of nanoAu, reducing graphene oxide by hydrazine at 100 °C for over 24 h [12]. Lam et al. reported a dopamine electrochemical biosensor based on the electrodeposition of PPy and nanoAu on chemical etching-graphene modified electrode [13]. More recently, Tiwari et al. reported a chemical-electrochemical route for the synthesis of a PPy, graphene oxide, and nanoAu nanocomposite [14]. First, a nanoAu–graphene sheet was fabricated using a chemical method, with two reduction agents sodium citrate dehydrate and sodium borohydrate at 100 °C. Second, a PPy-decorated nanoAu–graphene sheet was electropolymerized using a potentiodynamic method, and was used as a genosensor with good sensitivity and linearity. The reported PPy/RGO/nanoAu composites were prepared by chemical or chemical–electrochemical routes. In general, chemical methods have the advantages of large-scale and high yield, but the chemical process is not only time-consuming, but also uses excessive reducing agents which will leave lots of chemical residues, resulting in contaminated nanocomposites [15,16]. On the other hand, it is rather difficult to obtain relatively pure chemically reduced graphene oxide [17]. Therefore, exploring a more convenient, efficient, and greener route for the fabrication of a PPy–RGO–nanoAu biocomposite is still a challenge.

The key idea of this paper is to develop a facile eco-friendly one-step electrochemical method for the fabrication of a PPy–RGO–nanoAu biocomposite. In order to understand the electrochemical properties of the PPy–RGO–nanoAu biocomposite and its use as an electroactive surface modifying material, a hydrogen peroxide biosensor is further created and tested based on the as-prepared PPy–RGO–nanoAu modified glassy carbon electrode (GCE).

Hydrogen peroxide is known as the common form of reactive oxygen species in living cells, with an essential role as a mediator in a variety of biological processes, potentially capable of inducing various harmful biological modifications [18,19], and hydrogen peroxide is a crucial byproduct of highly selective oxidases commonly employed in biosensor design, such as glucose oxidase [20], cholesterol oxidase [21], and choline oxidase [22]. For example, glucose oxidase can catalyze glucose to the oxidized form—gluconolactone, and the reaction process is accompanied with the consumption of oxygen as well as the liberation of hydrogen peroxide [15]. Therefore, the accurate and rapid detection of hydrogen peroxide is not only important in monitoring and decoding relevant physiological pathways for hydrogen peroxide, but also very helpful in developing enzymatic-based biosensors for other analytes of interest [23,24]. Despite a staggering number of publications on hydrogen peroxide biosensor design, the market is still far from meeting many key end-user needs [25].

The proposed method enables the one-step electrochemical synthesis of PPy–RGO–nanoAu on the electrode surface without reducing agents commonly used in chemical methods. Several advantages of the study should be highlighted. First, it is attractive for PPy–RGO–nanoAu biocomposite synthesis due to its facile, simple, and green nature. Second, it can provide a facile eco-friendly one-step electrochemical approach to the fabrication of a variety of nanocomposites. Third, it can provide a potential electrochemical platform for the design of a variety of electrochemical biosensors. Advantageously, when hydrogen peroxide-producing oxidase enzymes are coupled with the as-prepared PPy–RGO–nanoAu, the method can be easily expanded to the detection of clinically relevant enzyme substrates, such as glucose, cholesterol, choline, and lactic acid.

## 2. Results

### 2.1. Preparation and Morphology Characterization

**Figure 1:**
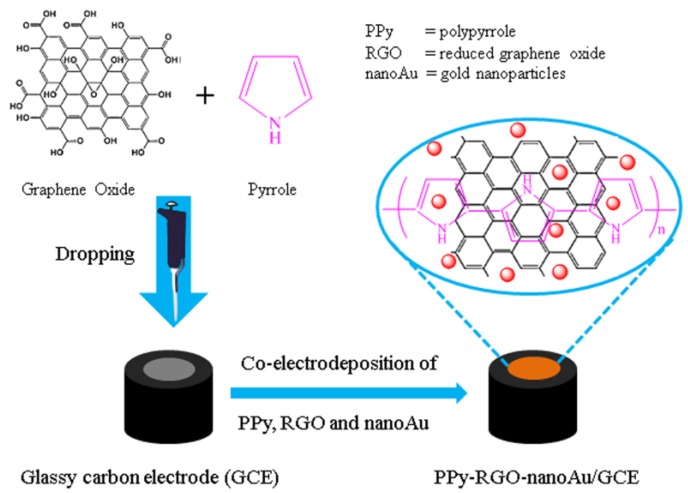
Schematic illustration of polypyrrole-reduced graphene oxide-gold nanoparticles biocomposite (PPy–RGO–nanoAu)-modified glassy carbon electrode (GCE).

The fabrication process of the polypyrrole-reduced graphene oxide-gold nanoparticles (PPy–RGO–nanoAu) modified glassy carbon electrode (GCE) is schematically illustrated in Figure 1. The mixture of graphene oxide and pyrrole monomer was firstly dropped onto the clean GCE surface. Then, the PPy–RGO–nanoAu biocomposite was one-step electrochemically synthesized by the electrooxidation of pyrrole monomer and the simultaneous electroreduction of graphene oxide and auric ions (Au^3+^) in aqueous solution using cyclic voltammetry. The obtained PPy–RGO–nanoAu consists of zero-dimensional gold nanoparticles, one-dimensional polypyrrole nanofibers, and two-dimensional reduced graphene oxide. Each component of PPy–RGO–nanoAu biocomposite is electroactive without non-electroactive substance. During the polymerization of pyrrole to form polypyrrole (either chemically or electrochemically), positive charges are created in its structure (polarons and bipolarons) [26]. Therefore, here PPy was not only used for conductive polymers, but also used as an effective linker for help in the immobilization of RGO and nanoAu on the electrode surface due to electrostatic interactions, π-π interactions [10,26,27], which contributes mechanical stability and proper conjugation to the nanohybrid composite [28]. The resulting PPy–RGO–nanoAu is expected to exhibit excellent material properties of parent components on the electrode surface, which may improve the charge transport properties, immobilization ability, and enhance signal responses due to the synergistic effect.

**Figure 2:**
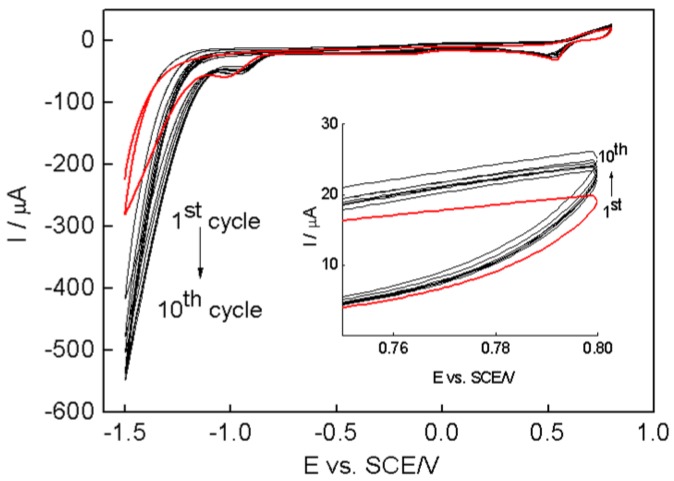
Typical cyclic voltammograms (CVs) of pyrrole/graphene oxide/GCE in HAuCl_4_ aqueous solution for 10 cycles with a scan rate of 25 mV/s. The scanning potential ranged from −1.5 V to 0.8 V vs. saturated calomel electrode (SCE). The arrows indicate the trend of redox current response during potential cycles.

Here we adopted an electrochemical co-depositing technique for the one-step synthesis of PPy–RGO–nanoAu composite without reducing agents. The electrochemical synthesis of conducting polymer PPy is more beneficial, offering facile precise control of the thickness and the structure of the resulting film [29]. Additionally, electrodepositing nanoAu and RGO onto the electrode surface using cyclic voltammetry has also been reported [15,30,31]. Within the electrodeposition process, PPy can be over-oxidized at high positive anodic potential to produce overoxidized PPy, which will decrease the conductivity and adherence at the electrode surface [13,32]. In this context, the co-electrodepositon of pyrrole, graphene oxide, and auric ions (Au^3+^) was performed by cyclic voltammetry in the potential ranging from −1.5 V to 0.8 V vs. a saturated calomel electrode (SCE) with a scan rate of 25 mV/s in HAuCl_4_·4H_2_O aqueous solution for 10 cycles, as shown in Figure 2. The potential of 0.8 V vs. SCE is not only sufficient to allow the formation of the PPy polymer, but can also avoid the over-oxidation of the PPy [33]. It can be seen that the large reduction current should be due to graphene oxide and auric ions, since the reduction of water to hydrogen occurs at more negative potentials [16,34,35]. The electrochemical reduction of graphene oxide could be attributed to the reduction of the functional groups such as –OH and –COOH on the graphene oxide surface [36,37], which will eliminate oxygenated defect sites and improve its electric properties [31]. The persistent redox current increasing with successive potential cycles indicates the electrodeposition of the PPy, RGO and nanoAu biocomposite was achieved on the surface of the glassy carbon electrode [38].

**Figure 3:**
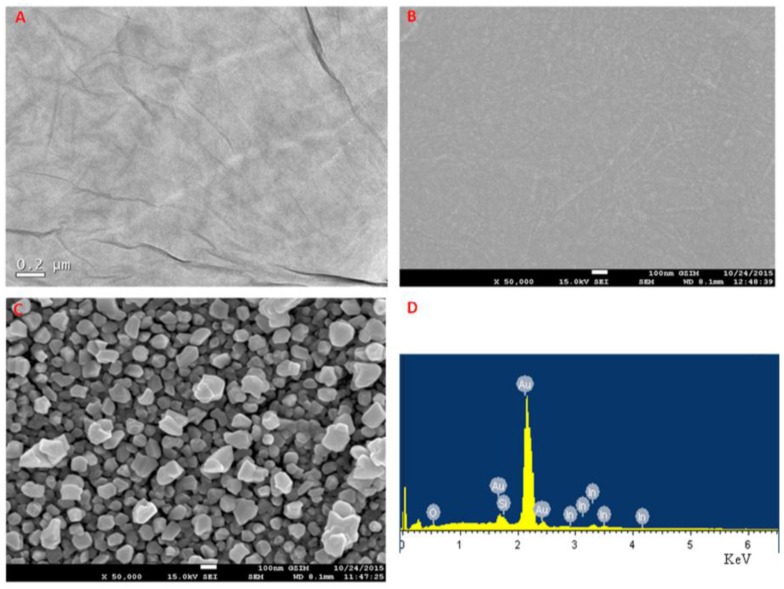
(**A**) Typical transmission electron microscopy image of graphene oxide; (**B**) scanning electron microscopy images of pyrrole/graphene oxide; and (**C**) PPy–RGO–nanoAu; and (**D**) energy dispersive spectroscopy spectrum of PPy–RGO–nanoAu.

A typical transmission electron microscopy image of graphene oxide and scanning electron microscopy images of pyrrole/graphene oxide and PPy–RGO–nanoAu are presented in Figure 3. It is shown that graphene oxide nanosheets are transparent thin flake-like shapes. The pyrrole/graphene oxide film shows a wrinkled pattern. After the co-electrodeposition of pyrrole monomer, graphene oxide, and auric ions, the obtained PPy–RGO–nanoAu composite film shows significantly rougher morphology than the pyrrole/graphene oxide film. It is obvious that some different nanostructures are seen as very tiny nanoparticles, due to the electrochemical reduction of auric ions to the formation of gold nanoparticles with average diameters less than 200 nm on the surface of PPy–RGO. The obtained morphologies are in close agreement with previous work in the literatures [15,39]. At the same time, the energy dispersive spectroscopy spectrum of PPy–RGO–nanoAu biocomposite is recorded in Figure 3D, confirming the presence of Au elements in the nanocomposites.

### 2.2. Electrochemical Behaviors of the PPy–RGO–NanoAu

**Figure 4:**
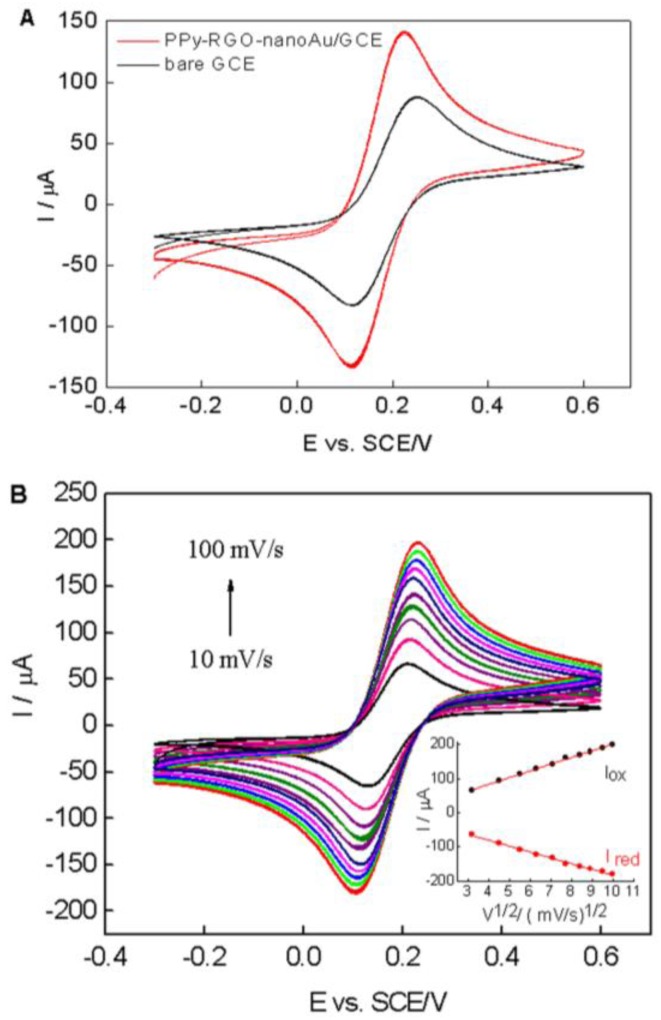
(**A**) CVs of the bare GCE (black line) and PPy-RGO-nanoAu/GCE (red line) in 0.1 M KCl containing 5 mM [Fe(CN)_6_]^3−/4−^ (1:1) with a scan rate of 50 mV/s; (**B**) CVs of the PPy-RGO-nanoAu/GCE recorded at 10, 20, 30, 40, 50, 60, 70, 80, 90 and 100 mV/s in 0.1 M KCl containing 5 mM [Fe(CN)_6_]^3−/4−^ (1:1). Inset: linear dependence of anodic and cathodic peak currents (*I*_ox_ and *I*_red_) vs. the square-root of the scan rate.

Cyclic voltammetry of [Fe(CN)_6_]^3−/4−^ is a valuable tool for testing the changes of electrode behavior after each assembly step of the modified electrode [40]. Figure 4A shows the cyclic voltammograms of the bare electrode (black line) and PPy–RGO–nanoAu/GCE (red line) in 0.1 M KCl containing 5 mM [Fe(CN)_6_]^3−/4−^ (1:1) with a potential ranging from −0.3 V to 0.6 V vs. SCE at a scan rate of 50 mV/s. A pair of redox peaks corresponding to the redox reaction of [Fe(CN)_6_]^3−/4−^ was observed at the bare GCE electrode (black curve). When the electrode was modified with PPy–RGO–nanoAu, the peak currents increased, and the peak-to-peak separation decreased (red curve), indicating the enhancing effect of PPy–RGO–nanoAu biocomposite on the electric conductivity.

According to the Randles–Sevcik equation [41]:

$$I_{p}=2.69\times 10^{5}AD^{1/2}n^{3/2}\gamma^{1/2}C$$

where *I*_p_ is the peak current (A), *A* is the electroactive area (cm^2^), *D* is the diffusion coefficient of the molecule in solution (cm^2^·s^−1^), *n* is the number of electrons involved in the redox reaction, γ is the potential scan rate (V/s), and *C* is the concentration of [Fe(CN)_6_]^3−/4−^ solution (mol·cm^−3^). In the equation, *D*, *n*, γ and *C* are constant parameter values, and the *I*_p_ value is linear with the electroactive surface area (A) [3]. Compared to the bare GCE, the *I*_p_ value of the PPy–RGO–nanoAu/GCE showed an obvious increase. The electroactive surface area of the PPy–RGO–nanoAu/GCE is about 1.63 times larger than bare GCE electrode, indicating that the presence of PPy, RGO, and nanoAu promote an increase in the electroactive area.

Figure 4B displays with increasing scan rates from 10 to 100 mV/s an increase in both the anodic and cathodic peak currents of the PPy–RGO–nanoAu/GCE. A good linearity of the peak current versus the square root of scan rate was obtained, as shown in the inset of Figure 4B. Two linear relationships were obtained with the regression equations of *I*_ox_ (μA) = 4.09 + 19.6*v*^1/2^(mV/s)^1/2^ (*n* = 10, *R*^2^ = 0.998), and *I*_red_ (μA) = −12.6 − 17.1*v*^1/2^(mV/s)^1/2^ (*n* = 10, *R*^2^ = 0.995), indicating a diffusion-controlled process.

### 2.3. Determination of Hydrogen Peroxide Using the PPy–RGO–NanoAu/GCE

**Figure 5:**
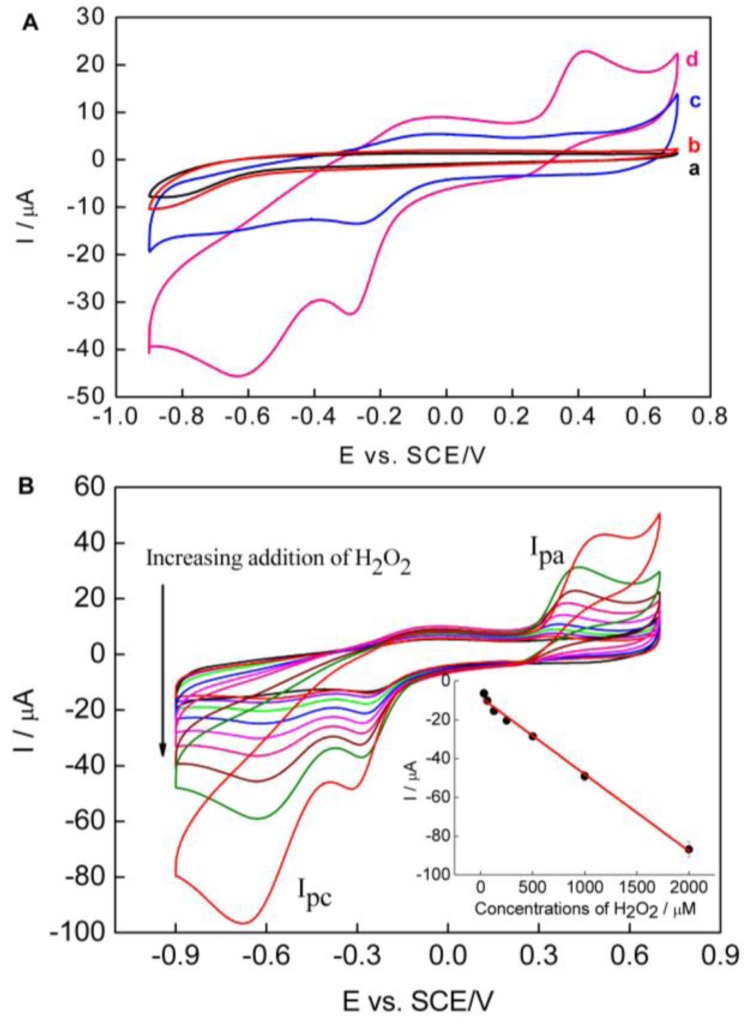
(**A**) CVs obtained at bare GCE (a,b) and PPy–RGO–nanoAu/GCE (c,d) in the absence (a,c) and the presence of 1 mM H_2_O_2_ (b,d); (**B**) CVs recorded at PPy–RGO–nanoAu/GCE in phosphate-buffered saline (PBS) containing different concentrations of H_2_O_2_ (0 μM–2 mM). Inset shows the linear dependence of *I*_pc_ over H_2_O_2_ concentrations (32 μM–2 mM). Error bars = ±standard deviation and *n* = 5. Scan rate: 50 mV/s.

The electrocatalytic activity of PPy–RGO–nanoAu/GCE toward H_2_O_2_ was studied by changing H_2_O_2_ concentration using cyclic voltammetry. Figure 5A shows the CVs of bare GCE and PPy–RGO–nanoAu/GCE in 0.1 M pH 7.4 phosphate-buffered saline (PBS) in the absence or presence of 1 mM H_2_O_2_ with the potential ranging from −0.9 V to 0.7 V vs. SCE at a scan rate of 50 mV/s, respectively. There is no obvious peak response observed at bare GCE and PPy–RGO–nanoAu/GCE in the absence of H_2_O_2_, indicating that the bare GCE and PPy–RGO–nanoAu/GCE are potentially inactive in this potential window [42]. It can be seen that a well-defined redox peak is observed with PPy–RGO–nanoAu/GCE in the presence of H_2_O_2_. The cathodic peak current is centered around −0.62 V vs. SCE, and the anodic peak current is centered around 0.41 V vs. SCE. No redox peak was observed at bare GCE under the same experimental conditions. The redox activity of H_2_O_2_ at the PPy–RGO–nanoAu/GCE can be ascribed to the good electrocatalytic activity of PPy–RGO–nanoAu biocomposite towards H_2_O_2_, which indicated that the as-prepared PPy–RGO–nanoAu is a highly electroactive matrix, and also confirms the role of PPy, RGO, and gold nanoparticles in the performance of the PPy–RGO–nanoAu-modified GCE.

Figure 5B displays the CVs of the PPy–RGO–nanoAu/GCE in 0.1 M pH 7.4 PBS containing different H_2_O_2_ concentrations. Both the oxidation peak current (*I*_pa_) and the reduction peak current (*I*_pc_) increase significantly upon increasing the concentration of H_2_O_2_. Compared to the current of *I*_pa_, the *I*_pc_ current is much higher. On the other hand, the interfering effect of common electroactive species will be limited at a negative applied potential. So, the current of *I*_pc_ at −0.62 V vs. SCE was chosen for quantitative determination of H_2_O_2_. It was also observed that the reduction peak potential negatively shifts with increasing concentration H_2_O_2_, and the reduction peak becomes broader, which is in agreement with previous studies [43]. The cathodic peak current at −0.62 V vs. SCE for PPy–RGO–nanoAu/GCE in 0.1 M pH 7.4 PBS without hydrogen peroxide is used as the background current. The cathodic peak currents at −0.62 V vs. SCE for PPy–RGO–nanoAu/GCE for different concentrations of hydrogen peroxide minus the background current was the current used for the calibration curve. The calibration curve was *I*(μA) = −0.04*C* (H_2_O_2_, μM) − 8.5, which is a linear relationship between the *I*_pc_ current and H_2_O_2_ concentration in the range of 32 μM–2 mM, with a correlation coefficient *R*^2^ of 0.995. From the slope of the calibration curve, the sensitivity of the PPy–RGO–nanoAu/GCE is calculated to be 40 μA/mM. The limit of detection (LOD) was found to be 2.7 μM at a signal-to-noise ratio of three. Relative standard deviation (R.S.D.) varied from 2.3% to 6.5% (*n* = 5) for the determination of hydrogen peroxide in the concentration range of 32–2000 μM.

### 2.4. Stability and Selectivity of the PPy–RGO–NanoAu/GCE

To verify the stability of the PPy–RGO–nanoAu biocomposite as a hydrogen peroxide biosensor, PPy–RGO–nanoAu/GCE was examined to 1 mM hydrogen peroxide every day in pH 7.4 PBS with the potential ranging from −0.9 V to 0.7 V vs. SCE at a scan rate of 50 mV/s, and the reduction peak current at −0.62 V vs. SCE was recorded. It still retained 94% of its initial current response after one month. The acceptable stability of the biosensor could be mainly attributed to the good stability of parent components. Notably, PPy is an effective linker for help in the immobilization of RGO and nanoAu on the electrode surface [10,26,27,28]. Moreover, the RGO coating is stable as a result of its poor solubility in common solvents [38].

**Figure 6:**
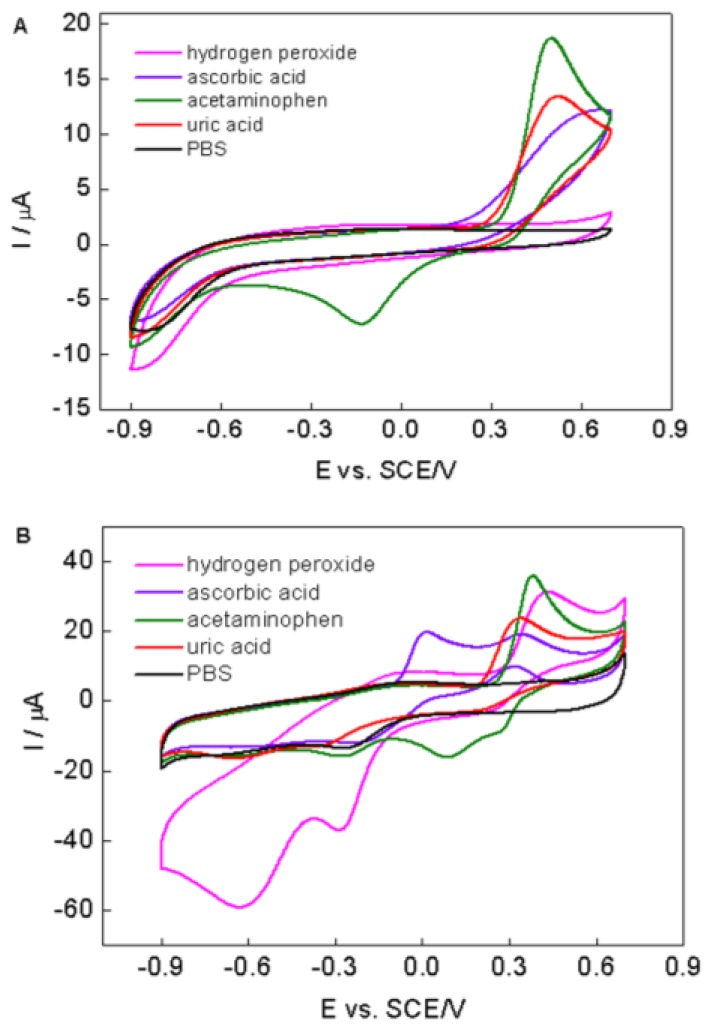
CVs of (**A**) bare GCE and (**B**) PPy–RGO–nanoAu/GCE in PBS containing 1 mM uric acid, 1 mM ascorbic acid, 1 mM acetaminophen, and 0.5 mM H_2_O_2_, respectively. Scan rate: 50 mV/s.

The selectivity of the biosensor was determined in the presence of potential interfering substances (such as uric acid, ascorbic acid, and acetaminophen) normally existing with H_2_O_2_. Figure 6 shows cyclic voltammetric responses of bare GCE (A) and PPy–RGO–nanoAu/GCE (B) in 0.1 M PBS with 1 mM uric acid, 1 mM ascorbic acid, 1 mM acetaminophen, and 0.5 mM H_2_O_2_ at the scan rate of 50 mV/s. As can be seen, obvious peak currents are found for uric acid, ascorbic acid, and acetaminophen at both bare GCE and PPy–RGO–nanoAu/GCE at positive potential (>0.2 V vs. SCE). While at negative potential (ca. −0.6 V vs. SCE), an obvious reduction response of hydrogen peroxide was only observed at PPy–RGO–nanoAu/GCE; however, the current responses of both bare GCE and PPy–RGO–nanoAu/GCE for the mentioned electroactive interfering species are all quite negligible. Although the concentration of H_2_O_2_ was two times less than interfering species, the PPy–RGO–nanoAu/GCE current response of H_2_O_2_ at −0.62 V vs. SCE was far higher than interfering current. Additionally, the concentrations of the interfering species used in this study are much greater than their normal physiological values [9,44,45]. The results clearly demonstrate that PPy–RGO–nanoAu/GCE has a good selectivity towards H_2_O_2_ at the negative potential, where possible interfering reactions are minimized. Furthermore, all current responses of the PPy–RGO–nanoAu/GCE are much better than those of the bare GCE, which also confirm the role of PPy–RGO–nanoAu in the overall performance of the PPy–RGO–nanoAu-modified GCE. All of the above revealed the potential application of PPy–RGO–nanoAu in electrochemical biosensors.

## 3. Materials and Methods

### 3.1. Reagents

All of the reagents were of analytical grade and used without further purification. Hydrogen peroxide (30%) was purchased from Guangzhou Chemical Reagent Factory (Guangzhou, China). Graphene oxide was purchased from Nanjin Xianfeng NANO material Tech Co., Ltd. (Shanghai, China). HAuCl_4_·4H_2_O and pyrrole monomer were purchased from J & K Scientific Ltd. (Beijing China). Alumina (diameter 0.1 and 0.05 μm) was purchased from Chen Hua Instruments (Shanghai, China). All experiments were performed at room temperature, approximately 25 °C.

### 3.2. Apparatus and Measurements

Cyclic voltammetry was carried out with CHI800C electrochemical analyzer with a conventional three-electrode system (Chen Hua Instruments, Shanghai, China), in which PPy–RGO–nanoAu modified glassy carbon electrode (GCE), a saturated calomel electrode (SCE) and a platinum wire electrode served as the working, reference, and auxiliary electrodes, respectively. Transmission electron microscopy of graphene oxide was carried out on a JEM-2100HR TEM at 200 kV (Tokyo, Japan). The surface morphological features of pyrrole/graphene oxide and PPy–RGO–nanoAu were characterized using JSM-7600F scanning electron microscopy (Tokyo, Japan).

### 3.3. Preparation of PPy–RGO–NanoAu/GCE

Glassy carbon electrode (3 mm in diameter) was chosen to act as the base electrode. The surface of the GCE electrode was polished using alumina (diameter 0.1 and 0.05 μm) slurries followed by thorough washing with water. The clean GCE was sonicated in ethanol and distilled water to remove adsorbed particles. A suspension of graphene oxide (10 μL, 0.2 mg/mL), and pyrrole monomer (0.3 M) was dropped onto the clean GCE surface and dried. After drying, the graphene oxide–pyrrole modified GCE was washed thoroughly and immersed into PBS containing 6.5 mM HAuCl_4_. Next, a continuous cyclic voltammetric sweep of 10 cycles with potential ranging from −1.5 V to 0.8 V vs. SCE was performed at a scan rate of 25 mV/s in order to obtain the PPy–RGO–nanoAu biocomposite on the GCE. The resulting PPy–RGO–nanoAu/GCE was stored at 4 °C in a refrigerator when not in use.

## 4. Conclusions

In this paper, we demonstrated a facile eco-friendly one-step electrochemical synthesis approach for the fabrication of a PPy–RGO–nanoAu biocomposite. Each component of PPy–RGO–nanoAu is electroactive without non-electroactive substance. As a highly electroactive matrix, a PPy–RGO–nanoAu-modified GCE was developed for the accurate and rapid electrochemical detection of hydrogen peroxide at −0.62 V vs. SCE, where possible interfering reactions are all quite negligible. Although there is still plenty of space for further optimization studies of both the PPy/RGO/nanoAu preparation and its application in electrochemical biosensors, the use of PPy–RGO–nanoAu as a matrix—along with the electrocatalytic detection of hydrogen peroxide—would provide a useful avenue for the preparation of chemical-modified electrodes and offers great promise for amperometric biosensors, especially those where the determination of the substrate is based on the electrochemical oxidation of hydrogen peroxide produced in a reaction catalyzed by an oxidase.
